# Effect of a Digital Literacy Program on Older Adults’ Digital Social Behavior: A Quasi-Experimental Study

**DOI:** 10.3390/ijerph191912404

**Published:** 2022-09-29

**Authors:** Hocheol Lee, Joo-Aeh Lim, Hae-Kweun Nam

**Affiliations:** 1 Yonsei Healthy City Research Center, Wonju 26493, Korea; 2 Department of Health Administration, Graduate School, Yonsei University, Wonju 26493, Korea; 3 Department of Preventive Medicine, Wonju College of Medicine, Yonsei University, Wonju 26426, Korea

**Keywords:** digital literacy education, smartphone usage, social isolation, online welfare services, social prescribing, aging population

## Abstract

In South Korea, digital literacy education programs are expected to help its older population participate in online welfare services to increase their social support, self-esteem and well-being. This quasi-experimental study assesses the effects of digital literacy education on digital device usage among rural-dwelling adults aged 65 and above and evaluates the positive effects of digital literacy education on depression, happiness, quality of life, self-efficacy and cognitive function. A digital literacy education program and a customized questionnaire survey were conducted to evaluate smartphone use competency and the program’s effects, respectively. We also conducted a chi-square test, paired *t*-test and difference-in-differences regression analysis. The experimental group showed a significant increase in smartphone usage and video recording capacity than the control group. The happiness and cognitive function scores for dementia screening increased significantly by a mean of 3.7 and 1.1 points, respectively, after digital literacy education. Cognitive function increased significantly by 1.305 points in the experimental group compared to the control group (β = 1.305, *p* = 0.05 *). Digital literacy education for older adults in rural areas greatly increased smartphone use, video recording capacity, happiness and cognitive function. Based on these findings, it is recommended that the government should implement digital literacy education for older adults in rural areas to increase their happiness and cognitive function.

## 1. Introduction

The development of medical technology has led to an increase in life expectancy and subsequent worldwide population aging. It is estimated that the number of people over the age of 65 will increase more than two-fold, from 730 million in 2019 to 1.5 billion in 2050 [1]. In this era, South Korea is expected to have the highest global aging rate of older people, with approximately 23% of the population over the age of 65 by 2050 [2]. South Korea became an aged society in 2017 and is expected to become a super-aged society in 2025 [3]. By 2035, people over 65 are expected to account for more than 30% of the total population. In 2020, 40% of Korean cities, counties and districts became super-aged societies [3].

Aging has led to social issues such as social isolation and depression in older adults as suggested by the World Health Organization (WHO) [4]. To curb the spread of the coronavirus disease 2019 (COVID-19) pandemic, declared by the WHO on 11 March 2020, the Korean government implemented quarantine measures such as social distancing, restricted travel outside residence and closure of social welfare facilities. As a result, older adults experienced social isolation leading to depression and poor health conditions such as heart disease, stroke and coronary artery disease. These in turn increased the mortality rate by 26–50% [5].

The WHO has suggested digital health (welfare services using digital devices) as an alternative to prevent the social isolation of older adults [6]. As social activities were restricted owing to COVID-19, various meetings and welfare programs were held online, which led to the expansion of the digital health market. The government’s COVID-19 guidelines, information and news were delivered through smartphones; communication with family, friends and acquaintances continues to occur mainly through smartphones. With such advances in digital technology, digital literacy among various classes of populations has emerged as a social issue. Digital literacy is defined as the ability to comprehend information generated by a digital device and perform tasks accordingly [7]. However, approximately 46.3% of older adults in Korea are unfamiliar with digital devices and feel burdened using them [8]. The percentage of older adults aged 65 and above who are not comfortable using digital devices was 34% in England, 29% in the US, 18% in Japan, 21% in Russia and 27% in Germany, which were lower than that of South Korea. In contrast, the percentage was 47% in Spain, 48% in Mexico, and 50% in Argentina, which were higher than that of South Korea [9].

A digital gap arises between generations because older adults have limited knowledge and ability to utilize digital devices [10]. In particular, older adults are known to experience challenges in certain tasks when using the internet and smartphones, such as in entering, selecting and reading text [11].

The digital illiteracy rate is lower in rural areas than that in urban areas, leading to a higher sense of digital isolation. This has subsequently led to a continuous increase in the digital divide between older adults in rural and urban areas [12].

To reduce this gap in the digital competence of the older adult population and improve their digital literacy, the South Korean government launched a digital platform government on 1 July 2022. This digital platform government has nine basic principles, which include gradually converting public services to digital platforms and merging public services with the internet and smartphone applications. Although the digital platform government is expected to make the use of public services convenient, enhancing digital literacy in a social group that is vulnerable to digital technology, such as older adults, has been presented as an ongoing challenge [13].

Recently, medical and public institutions, including the Ministry of Health and Welfare, are providing various information on dementia, hospitals, diseases and health to older adults through smartphone applications [14]. In particular the “Dementia Check Application” by the Ministry of Health and Welfare, “Dementia Books Application” by the Korean government, “Dementia Prevention Brain Game Application” by Samsung Seoul Hospital and “AI Personal Care Robot” digital health care by local governments illustrate the demand for digital literacy and the importance of smartphone usage. To keep up with the trend, the government must expand education for older adults on smartphone use, and this may subsequently lead to happiness, prevention of dementia and increased access to information.

Digital literacy education for older adults who are unfamiliar with digital device usage has increased participation in online welfare programs and frequency of communication with family and acquaintances, leading to increased social support and self-esteem [15]. Subsequently, mental as well as physical health has improved and participation in welfare programs using digital devices for more than two years had preventive effects against dementia in older adults.

We aimed to, first, assess the effects of digital literacy education on digital device usage in older adults aged 65 and above living in rural areas. Second, we evaluated the positive effects of digital literacy education on depression, happiness, quality of life, self-efficacy and cognitive function among older adults. Third, we attempted to provide a basic framework for national policies on digital health care for older adults using implications of digital literacy education.

## 2. Materials and Methods

### 2.1. Study Design

A quasi-experimental design study using the difference-in-differences approach was conducted to investigate the effects of digital literacy education on smartphone usage competency, happiness, subjective health, depression, quality of life, self-efficacy and cognitive function in older adults over the age of 65 living in rural areas.

The participants of this study were from the older adult population (over the age of 65) living in Wonju-si, Gangwon-do, Korea. The total older adult population in this area is 53,390, which accounts for 15% of the total population [16]. The sample size was calculated using GPower 3.1 software, with an F-test (linear multiple regression fixed model), with effect size of 0.15, power of 0.95 and *p*-level of 0.05. The minimum sample size obtained was 107.

### 2.2. Participants

This study was conducted in collaboration with an older adult center in Wonju-si, Gangwon-do province. Therefore, a total of 62 people over the age of 65 who volunteered to attend digital literacy education in Wonju-si were included in this study. In addition, another group comprising 62 older adults who did not wish to receive digital literacy education and were part of the older adult center of Wonju-si was selected as the control group. However, at the end of the survey, 45 people in the experimental group and 36 people in the control group responded to the survey; the remaining participants were unable to participate on the last day due to COVID-19, and hence, the survey was not conducted.

### 2.3. Digital Literacy Education

A digital literacy education program was conducted to evaluate smartphone usage competency in older adults. Two smartphone instructors, affiliated to the Wonju Welfare Center, conducted the program across a total of five locations including Y University Healthy City Research Center, the Wonju Senior Center and small libraries. The official name of the program was “Social prescribing through digital literacy education for older adults in Wonju-si”. A total of 62 older adults participated in the program once a week for six weeks from 25 October 2021 to 3 December 2021 (Table 1).

### 2.4. Variables

To assess the effects of the digital literacy education program, a standard questionnaire was used taking into account the local conditions of Wonju-si. The questionnaire included participant characteristics, happiness, subjective health, depression, quality of life, self-efficacy and a cognitive function test.

Participant characteristics included gender, education level, age, residence type and smartphone usage. Happiness with current life was scored out of 100 points. Subjective health was based on evaluated health status in the previous month and was scored out of 100 points. Furthermore, the ability to use a smartphone was coded as “capable” or “no phone calls” for using smartphones for phone calls, taking photos and video recording.

Depression was evaluated using the Geriatric Depression Scale Korean Version (GDS-K). The GDS-K is one of the most widely used instruments for assessing and screening for depression among older adults. It is a 30-item tool and was validated for use in older adults in Korea [17]. Quality of life was assessed using the EuroQol-5 dimension (EQ-5D) developed by the EuroQol Group. This tool comprises five items (mobility, self-care, usual activities, pain/discomfort and anxiety/depression) rated on a five-point scale. The tool was validated for use in Korea [18].

Self-efficacy was assessed using the 10-item General Self-Efficacy Scale (GSE) [19,20]. Each item is rated on a four-point Likert scale, and a higher score indicates higher self-efficacy.

Additionally, a cognitive function test was conducted using the Korean-mini mental state examination (K-MMSE), a Korean-modified, translated and validated version of the MMSE developed by Folstein in 1975 [21]. The total possible score on the K-MMSE is 30.

### 2.5. Data Analysis

SPSS 26.0 was used for statistical analysis of the effects of the digital literacy education program. First, a Pearson chi-square test and independent two-sample *t*-test were conducted to analyze key indicators of changes pre- and post-education in the experimental group. Second, to understand the effects of key indicators in the experimental group, difference-in-differences regression analysis was conducted with the control group. The regression analysis compared the differences pre- and post-education between the two groups and assessed the effects of the program in consideration of natural changes and effects from external factors.

### 2.6. Ethical Approval

All components of this survey were approved by the institutional review board (IRB) of Yonsei University in Korea (IRB document number: 1041849-202112-SB-207-02). Written informed consent was obtained from all respondents prior to the data collection. Additionally, we emphasized the respondents’ right to refuse the survey request on the first page of the online survey form.

## 3. Results

This section may be divided by subheadings. It should provide a concise and precise description of the experimental results and their interpretation, as well as the experimental conclusions that can be drawn.

### 3.1. Participant Characteristics

The demographic characteristics of the participants are shown in Table 2. The experimental group included 19 male participants (30.6%) and 43 female participants (69.4%) and showed the same distribution of sex as the control group (χ^2^ = 3.466, *p* = 0.063). There were 3 participants with no education (4.8%), 15 with an elementary school certificate (24.2%), 10 who graduated from middle school (16.1%) and 34 who graduated from high school or higher (54.8%) in the experimental group. For residence type, 24 participants of the experimental group lived alone (38.7%), 28 lived with a spouse (45.2%), 5 lived with children (8.1%), 4 lived with a spouse and children (6.5%) and 1 (1.6%) had another residence type. The distribution of residence type was the same between the experimental and control groups (χ^2^ = 1.934, *p* = 0.748). The mean age of the experimental group was 75.2 years and was similar to that of the control group (t = −0.795, *p* = 0.428). 

### 3.2. Comparison of Pre- and Post-Digital Literacy Education Changes

Changes in post-digital literacy education were assessed in the experimental and control groups in Table 3. The frequency of phone calls made using smartphones by the older adults in the experimental group increased significantly by 8.5% after education (t = 1.934, *p* = 0.026 *). In contrast, no changes were observed in the control group. 

### 3.3. Difference-in-Differences Regression

To understand the effects of digital literacy education, difference-in-differences regression analysis was conducted (Table 4). Cognitive function increased significantly by 1.305 points in the experimental group compared to the control group (β = 1.305, *p* = 0.05 *).

The F-test was conducted to examine the goodness-of-fit of the difference-in-differences model. The *t*-test results were happiness *p* = 0.072, subjective health *p* = 0.062, depression *p* = 0.049, quality of life *p* = 0.077, self-efficacy *p* = 0.018, and cognitive function *p* < 0.000.

## 4. Discussion

In this study, the effects of digital literacy education on depression, happiness, quality of life, self-efficacy and cognitive function were assessed in older adults over the age of 65 living in rural areas. Digital literacy differs from the concept of literacy. The generally accepted definition of literacy pertains to social customs, reading and writing [22]. However, as a result of the diversified social conditions and customs, social practices that need to be identified and discussed have also become diversified. Particularly, a recent definition of literacy has expanded the scope of literacy to the understanding and communication in visual, electronic and digital forms [23]. Digital literacy is an important construct amid increased delivery and communication of various messages socially through digital devices. In other words, people with a poor understanding and competency of digital literacy in the present era of continuous advances in digital technologies will be marginalized from social communication [24]. Digital literacy is particularly low among older adults aged 65 years and over, in addition to their poor access to digital devices. Increasing digital literacy will enable various forms of communication in society, and communication was previously documented to have positive effects on depressive mood, happiness in life, quality of life, self-efficacy and cognitive functions [25]. Therefore, this study examined digital literacy education in relation to depressive mood, happiness in life, quality of life, self-efficacy and cognitive functions.

First, upon assessment of the participant demographics, there were more female (43, 69.4%) than male (19, 30.6%) participants. According to a previous study, a female older adult’s brain was more active when sending vital signs in external and social activities than that of a male older adult [26,27]. Consistent with this finding, there was a higher number of females who volunteered to participate in this study.

Participants in the digital literacy education program showed significant increases in smartphone use and video recording capacity compared to the control group. This finding supports that the digital literacy education for six weeks increased smartphone usage in the older adults living in rural areas. According to previous studies on older adults in Korea, using smartphones positively affected satisfaction with life, satisfaction with living and social isolation in older adults [28,29,30,31]. Consistent with previous findings, the digital literacy education program provided in this study increased older adults’ capacity to use smartphones, through direct and indirect effects on various indicators. In fact, the happiness and cognitive function scores for dementia screening increased significantly by a mean of 3.7 points and 1.1 points, respectively, after the digital literacy education. This demonstrates that smartphone education for older adults in rural areas is effective not only in teaching them how to use the smartphones but is also effective in preventing dementia and promoting happiness. However, the control group showed a self-efficacy score that significantly increased by 4.5 points compared to the baseline, as shown by a *t*-test. The control group demonstrated a greater change from the baseline than the experimental group, who only showed a change in self-efficacy score by 1 point. We speculated this was affected by several exogenous variables. According to a study validating the GSE, repeated administration of a questionnaire causes participants to become familiarized with the scale, which may result in high self-efficacy in the data obtained from repeated questionnaire administration [32]. To specifically pinpoint the effects of the education in consideration of such exogenous variables, we conducted difference-in-difference regression analysis using data from the experimental and control groups.

In this study, the changes in the various indicators (i.e., happiness, subjective health, depression, quality of life, self-efficacy and cognitive function) made by intervention were analyzed using difference-in-differences regression analysis by minimizing non-sampling error and external factors. As a result, the cognitive function score for dementia screening increased significantly by 1.305 points post-education in the experimental group, more than in the control group. In agreement with our findings, previous studies have also shown that older adults with cognitive impairment who used smartphones showed improved prospective memory and cognitive function [33,34]. 

Several studies worldwide, including in Korea, have administered and evaluated digital literacy education interventions in older adults in an attempt to reduce the digital gap. According to a Japanese study that evaluated the effects of digital literacy education in older adults, participants showed an increase of self-efficacy after education. The said study provided a 12-h intensive program teaching about the internet and personal computers, and self-efficacy was assessed using a self-efficacy scale [35]. A Chinese study that evaluated the effects of a tablet computer intervention in older adults also observed increased social support after the intervention, and the older adults searched for and practiced health management after [36]. Compared to these studies, digital literacy education provided in Korea is expected to not only improve various functions in older adults but also promote continuous health management by increasing their access to health information. For the South Korean government to implement the digital platform government and data-based public service policies, digital literacy education will be an effective strategy to improve the digital competence of older adults, who are vulnerable to digital technology. 

Further, a systematic review of 27 studies on digital literacy reported that digital literacy interventions familiarized older adults to mobile devices as well as improving the protection of personal information, which secondarily led to the prevention of financial victimization [25].

Several limitations must be considered in the interpretation of this study’s findings. First, this study was conducted on a small number of people aged 65 and over living in Wonju-si, Gangwon-do. In particular, the control group was more educated than the experimental group at the baseline, and this may have caused an error in the analysis. Subsequent studies should take this into consideration by performing propensity matching or adding it as a control variable for the analysis. Thus, the results cannot be generalized for groups of different gender, economic level and age. Second, in this study, endline assessment was conducted immediately after six weeks of digital literacy education. For more accurate results in future studies, it would be necessary to determine the continuity of effects through a second endline assessment after several months. Third, among the demographic characteristics of participants, there was a significant difference in the education level between the experimental and control groups. There were limitations in recruiting participants during the COVID-19 pandemic, which may have led to the difference in the basic characteristics. In follow-up studies post COVID-19 pandemic, digital literacy education programs taking into account these limitations would be necessary.

## 5. Conclusions

In conclusion, digital literacy education for older adults in rural areas greatly increased smartphone use and video recording capacity, as well as happiness and cognitive function. In particular, compared to the control group, the experimental group showed significantly improved cognitive function. Based on these findings, the digital literacy education has positive effects on the cognitive function of rural-dwelling older adults, although further studies with larger samples and more areas are needed.

## Figures and Tables

**Table 1:** Digital literacy education curriculum.

Week	Education Topic	Content	Duration (Min)
1	Basic smartphone operation	‒ Baseline assessment ‒ App installation, connecting WI-FI, typing	60
2	Sending text messages	‒ Sending text messages (including voice messages) ‒ Saving and deleting contacts	60
3	Taking and sharing photos	‒ Taking pictures and saving them ‒ Sending photos to family, friends and acquaintances	60
4	Social App	‒ Installing and using social apps ‒ Communicating with family, acquaintances and friends through social apps	60
5	Search engine applications (Naver)	‒ Installing search engine applications ‒ Searching for news using keywords	60
6	Internet banking	‒ Installing internet banking apps ‒ Sending money to family, friends and acquaintances ‒ Endline assessment	60

**Table 2:** Demographic characteristics of participants.

	Experimental Group (n = 62)		Control Group (n = 62)		t/χ^2^	*p*-Value
	N	%	n	%		
Sex						
Male	19	30.6	10	16.1	3.466	0.063
Female	43	69.4	52	82.3		
Education level						
No education	3	4.8	15	23.3	10.160	0.017 *
Elementary school	15	24.2	16	24.2		
Middle school	10	16.1	10	16.1		
Highschool and above	34	54.8	21	33.9		
Residence type						
Living alone	24	38.7	21	35.0	1.934	0.748
Living with a spouse	28	45.2	32	53.3		
Living with children	5	8.1	3	5.0		
Living with a spouse and children	4	6.5	6	6.7		
Others	1	1.6	0	0.0		
Religion						
No religion	19	30.6	21	33.3	2.781	0.595
Christian	21	33.9	17	26.7		
Catholic	9	14.5	7	11.7		
Buddhist	12	19.4	17	28.3		
Others	1	1.6	0	0.0		
Age (M ± SD)	75.2 ± 5.2		76.1 ± 6.9		−0.795	0.428

* *p* < 0.05, ** *p* < 0.01, *** *p* < 0.001.

**Table 3:** Comparison of pre- and post-digital literacy education changes.

	Experimental Group			Control Group		
	Baseline (n = 62)	Endline (n = 45)	t/χ^2^ (*p*-Value)	Baseline (n = 62)	Endline (n = 36)	t/χ^2^ (*p*-Value)
Smartphone use for phone calls						
Capable	40 (64.5%)	37 (83.0%)	4.968 (0.026 *)	45 (72.6%)	30 (83.3%)	1.466 (0.226)
No phone calls	22 (64.5%)	8 (17.0%)		17 (27.4%)	6 (16.7%)	
Photos						
Capable	47 (75.8%)	40 (88.9%)	2.936 (0.087)	46 (74.2%)	22 (61.1%)	1.835 (0.176)
Not capable	15 (24.2%)	5 (11.1%)		16 (25.8%)	14 (38.9%)	
Video recording						
Capable	23 (37.1%)	26 (58.3%)	4.493 (0.049 *)	27 (43.5%)	13 (36.1%)	0.522 (0.527)
Not capable	39 (62.9%)	19 (41.7%)		35 (56.5%)	23 (63.9%)	
Happiness (M ± SD)	63.1 ± 19.1	66.8 ± 19.4	−1.036 (0.032 *)	66.0 ± 18.8	66.4 ± 22.2	−0.085 (0.933)
Subjective health (M ± SD)	60.9 ± 19.2	59.4 ± 19.3	0.403 (0.688)	56.2 ± 22.1	57.8 ± 22.2	−0.349 (0.728)
Depression (M ± SD)	10.4 ± 6.1	8.4 ± 5.4	1.738 (0.077)	10.9 ± 5.3	8.9 ± 5.9	1.769 (0.080)
Quality of life (M ± SD)	8.0 ± 3.3	7.4 ± 2.8	1.009 (0.315)	7.8 ± 2.8	7.9 ± 2.9	−0.214 (0.831)
Self-efficacy (M ± SD)	57.1 ± 9.5	58.1 ± 7.5	−0.631 (0.530)	55.6 ± 8.5	60.1 ± 11.2	−2.275 (0.025 *)
Cognitive function (M ± SD)	27.9 ± 1.9	29.0 ± 1.4	−3.487 (0.001 **)	27.3 ± 3.3	27.1 ± 3.4	0.274 (0.784)

* *p* < 0.05, ** *p* < 0.01, *** *p* < 0.001.

**Table 4:** Results of difference-in-differences regression analysis of the effects of digital literacy education.

Indicator	*β*	t	95% CI	*p*-Value
Happiness	3.371	0.611	[−7.499, 14.242]	0.542
Subjective health	−3.070	−0.529	[−14.499, 8.360]	0.597
Depression	0.058	0.036	[−3.140, 3.255]	0.972
Quality of life	−0.709	−0.852	[−2.348, 0.931]	0.395
Self-efficacy	−3.502	−1.382	[−8.499, 1.494]	0.169
Cognitive function	1.305	1.973	[0.001, 2.609]	0.050 *

* *p* < 0.05, ** *p* < 0.01, *** *p* < 0.001.

## Data Availability

Not applicable.
